# Caractéristiques cliniques et épidémiologiques des décès COVID-19 en Tunisie avant l’émergence des VOCs (mars 2020-février 2021)

**DOI:** 10.11604/pamj.2022.43.172.35544

**Published:** 2022-12-05

**Authors:** Sonia Dhaouadi, Aicha Hechaichi, Hajer Letaief, Mouna Safer, Emna Mziou, Khouloud Talmoudi, Chiraz Borgi, Henda Chebbi, Naoufel Somrani, Mohamed Belahj Ali, Souad Yahyaoui, Amal Mseddi, Mohamed Kouni Chahed, Mustapha Ferjani, Nissaf Bouafif-Ben Alaya

**Affiliations:** 1 Observatoire National des Maladies Nouvelles et Emergentes, Tunis, Tunisie,; 2 Faculté de Médecine de Tunis, Université Tunis El Manar, Tunis, Tunisie,; 3 Ministère de la Santé, Tunis, Tunisie,; 4 Direction Régionale de la Santé, Tunis, Tunisie

**Keywords:** COVID-19, mortalité, épidémie, surveillance épidémiologique, standardisation, Tunisie, COVID-19, mortality, epidemic, epidemiological surveillance, standardization, Tunisia

## Abstract

**Introduction:**

les objectifs de ce travail étaient de décrire le profil clinique et épidémiologique des décès COVID-19 en Tunisie notifié à l´ONMNE (Observatoire National des Maladies Nouvelles et Émergentes) entre le 02 mars 2020 et le 28 février 2021 et de comparer la mortalité COVID-19 enregistrée en Tunisie aux données internationales.

**Méthodes:**

nous avons mené une étude nationale descriptive longitudinale prospective auprès des données colligées à travers le système national de surveillance de l´infection au SARS-CoV-2 de l´ONMNE, Ministère de la Santé. Tous les décès COVID-19 survenus en Tunisie entre mars 2020 et février 2021 ont été inclus dans cette étude. Les données ont été colligées auprès des structures hospitalières, des municipalités et des directions régionales de la santé. Les notifications des décès ont été collectées à travers différentes sources d´information (méthode de triangulation): les directions régionales de santé, Le ShocRoom (Strategic Health Operations Center ou Centre stratégique d´opérations sanitaires), les structures sanitaires publiques et privés, la Cellule de Crise de la Présidence du Gouvernement, La Direction d´Hygiène et de protection de l´Environnement, le Ministère des Affaires Locales et de l´Environnement, dans le cadre de suivi des cas confirmés par l´équipe de l´ONMNE, les résultats RT-PCR/TDR positifs en post mortem.

**Résultats:**

durant la période de l´étude, 8051 décès ont été enregistrés soit une mortalité proportionnelle de 10,4%. L´âge médian était de 73 ans avec un intervalle interquartile de 17 ans. Le sex-ratio (M/F) était de 1,8. Le taux brut de mortalité était de 69,1/100 000 habitants et la létalité était de 3,5%. L´étude de la courbe épidémique a montré 2 pics de décès le 29 octobre 2020 et le 22 janvier 2021 avec respectivement 70 et 86 décès notifiés. La distribution spatiale des décès a montré que la région du Sud tunisien avait enregistré le taux de mortalité le plus élevé. Les patients âgés de 65 ans et plus étaient les plus concernés (73,7% des cas) avec un taux brut de mortalité de 570,9/100 000 habitants et une létalité de 13,7%.

**Conclusion:**

la stratégie de prévention basée sur les mesures de santé publique doit être renforcées par le déploiement rapide de la vaccination anti-COVID-19 surtout pour les populations à risque de décès.

## Abstract (en)

**Introduction:**

the purpose of this study was to describe the clinical and epidemiological features of COVID-19-related deaths in Tunisia notified at the ONMNE (National Observatory of New and emerging Diseases) between 2^nd^ March 2020 and 28^th^ February 2021 and to compare COVID-19-related deaths recorded in Tunisia with the international data.

**Methods:**

we conducted a national prospective longitudinal descriptive study of data collected from the National Surveillance System of SARS-CoV-2 infection of the ONMNE, Ministry of Health. All COVID-19-related deaths that occurred in Tunisia between March 2020 and February 2021 were included in this study. Data were collected from hospitals, municipalities and regional health departments. Death notifications were collected from multiple data sources (triangulation): The Regional Directorate of Basic Health Care, the ShocRoom (Strategic Health Operations Center), public and private health facilities, the Crisis Unit of the Presidency of the Government, the Directorate for Hygiene and Environmental Protection, the Ministry of Local Affairs and the Environment, as part of the follow-up of confirmed cases by the ONMNE team, positive RT-PCR / TDR post mortem results.

**Results:**

during this study, 8051 deaths were recorded, corresponding to a proportional mortality of 10.4%. The median age was 73 years, with an interquartile range of 17 years. Sex-ratio (M/F) was 1.8. The crude death rate was 69.1/100 000 inhabitants and fatality rate was 3.5%. The analysis of the epidemic curve showed 2 peaks of deaths on 29^th^ October 2020 and 22^nd^ January 2021, with 70 and 86 deaths notified respectively. The spatial distribution of mortality showed that the southern Tunisian region had the highest mortality rate. Patients aged 65 and over were most affected (73.7% of cases) with a crude mortality rate of 570.9/100,000 inhabitants and a fatality rate of 13.7%.

**Conclusion:**

prevention strategy based on public health measures must be reinforced by the rapid deployment of anti-COVID-19 vaccination, especially for people at risk of death.

## Introduction

Durant les 5 dernières décennies, le monde a été confronté à des épidémies causées par des virus émergents zoonotiques dont les coronavirus [1,2]. L´épidémie COVID-19 (Coronavirus Disease 2019) causée par le béta-coronavirus SARS-Cov-2 (*Severe Acute Respiratory Syndrome CoronaVirus 2*), initialement déclarée en Chine (Wuhan) s´est rapidement propagée à travers le monde pour causer une pandémie comme l´a déclaré l´OMS le 11 mars 2020, soit la première pandémie causée par un coronavirus [2]. Le virus a diffusé au niveau international dans un délai d'un mois après la première identification le 31 décembre 2020, témoignant d´une transmission interhumaine à travers le contact étroit interhumain [3,4]. A la date du 28 février 2021, soit près d´un an après la déclaration de la pandémie, le nombre cumulé de cas dans le monde a dépassé les 100 millions (113 472 187) dont 2 520 653 décès (2,2%) [5]. L´Amérique et l´Europe représentaient les épicentres de l´épidémie aussi bien pour les cas que pour les décès cumulés pendant la période d´étude [6]. La présentation clinique de la COVID-19 est hétérogène et non spécifique allant des formes asymptomatiques et bénignes aux formes sévères, critiques et au décès [7,8]. La transmission interhumaine à travers les gouttelettes respiratoires aussi bien par les sujets symptomatiques que par les asymptomatiques rend la propagation du virus plus rapide à l´échelle communautaire [4]. Le nombre de décès témoigne de la gravité de l´épidémie et considéré comme étant l´indicateur le plus fiable pour refléter la dynamique d´évolution de l´épidémie [9]. En effet, le nombre de décès pour 100 000 habitants (ou pour 1 million d´habitant) reflète à la fois le degré de gestion de l´épidémie ainsi que la qualité de prise en charge des cas. La Tunisie, comme le reste des pays du monde, n´a pas été épargnée de cette flambée. Depuis le premier cas enregistré le 02 mars 2020, 233 277 cas cumulés et 8051 décès ont été déclarés à l´Observatoire National des Maladies Nouvelles et Émergentes (ONMNE) - Ministère de la Santé à la date du 28 février 2021 [10]. La mortalité liée au COVID-19 dépend de plusieurs facteurs tels que la précocité du diagnostic, les caractéristiques socio démographiques, les antécédents du patient ainsi que la précocité et l´efficacité de la prise en charge thérapeutique. L´objectif principal de ce travail était de décrire les caractéristiques épidémiologiques et cliniques des décès COVID-19 en Tunisie afin de proposer des mesures de prévention. L´objectif secondaire était de comparer la mortalité liée au COVID-19 en Tunisie aux données internationales.

## Méthodes

**Type d´étude:** il s´agissait d´une étude nationale observationnelle descriptive type longitudinale avec un recueil prospectif des données.

**Population d´étude:** nous avons inclus tous les décès COVID-19 déclarés à l´ONMNE à travers les différentes sources d´information depuis l´enregistrement du premier cas COVID-19 en Tunisie (le 02 mars 2020) et jusqu´à la date du 28 février 2021, période caractérisée par la circulation prédominante des souches sauvages du SARS-CoV-2 en Tunisie.

**Définitions d´un décès COVID-19:** la définition adoptée à des fins de surveillance épidémiologique est basée sur des références internationales (Organisation mondiale de la santé, Centre européen de prévention et de contrôle des maladies (ECDC) et Centres pour le contrôle et la prévention des maladies (CDC) et nationale (ONMNE) [11,12].

### Classification d´un décès COVID-19

**Décès COVID-19 confirmé:** les décès avec confirmation par RT-PCR ou TDR-g.

**Décès COVID-19 probable:** les décès pour lesquels l´examen virologique ou moléculaire était négatif ou non fait mais présence de lésions scannographiques évocatrices.

### Critères d´éligibilité

**Critères d´inclusion:** tous les décès avec confirmation de la COVID-19 sans autre cause directe du décès ont été inclus. Les décès parmi les non-résidents permanents en Tunisie et les décès de nationalité autre que tunisienne survenus en Tunisie et dont le diagnostic a été fait en Tunisie, ont été aussi inclus.

**Critères de non inclusion:** nous n´avons pas inclus les décès des sujets déclarés guéris de COVID-19 avant le décès et les décès chez des patients COVID-19 positifs avec une autre cause directe de décès.

**Critères d´exclusion:** nous avons exclu les décès cliniquement suspects sans confirmation par un examen complémentaire (preuve moléculaire virologique, ou scannographique). Les sujets diagnostiqués et décédés de la COVID-19 en dehors de la Tunisie et enterrés dans le territoire tunisien ont été aussi exclus.

**Collecte et sources des données:** la COVID-19 est considérée une maladie à déclaration obligatoire en Tunisie depuis le 24 août 2020 (numéro de classification internationale R 1701.0) [13]. Afin de capturer tous les décès et de maximiser l´exhaustivité des déclarations, la surveillance épidémiologique des décès COVID-19 s´est basée sur la méthode de triangulation en croisant plusieurs sources d´information [14-16]. Les différentes sources d´informations incluaient : a) les directions régionales de santé; b) le ShocRoom (Strategic Health Operations Center ou Centre stratégique d´opérations sanitaires); c) les structures sanitaires publiques et privés; d) la Cellule de Crise de la Présidence du Gouvernement (CCPG); e) la Direction d´Hygiène et de protection de l´Environnement (DHMPE); f) le Ministère des Affaires Locales et de l´Environnement (Municipalités); g) dans le cadre de suivi des cas confirmés par l´équipe de l´ONMNE; g) les résultats RT-PCR /TDR positifs en post mortem.

Les déclarations sont transmises immédiatement et en urgence (dans les 24 heures) à l´aide d´une fiche de déclaration rapide de décès adressée par email et/ou par fax. Les informations recueillies sur la fiche de déclaration associent l´identité et les coordonnées du déclarant, le nom et le prénom du défunt, sa date de naissance, son genre, son gouvernorat de résidence ainsi que l´adresse exacte, la date de confirmation, le mode de confirmation, la date et le lieu de décès. Des données complémentaires sur les comorbidités sont aussi collectés lors de d´investigation systématique de tous les cas de décès auprès des déclarants ou après croisement avec la base des cas confirmés à l´ONMNE. Les données de la taille des populations, globalement, par âge, genre et gouvernorat pour l´année 2019 ainsi que la mortalité toutes causes confondues pour la même période de l´étude ont été fournies par l´Institut National de la Statistique (INS) [17,1,8]. Nous nous sommes référés aux données de la banque mondiale pour obtenir les données des populations par âge des autres pays [19].

**Analyse des données:** la saisie des données a été faite par le logiciel MS-Excel. L´analyse des données après vérification et nettoyage, a été réalisée de façon anonyme moyennant le logiciel Epi Info. Pour la description des variables quantitatives, nous avons calculé des moyennes avec des écarts types (±), des médianes avec l´intervalle interquartile (Q1;Q3) (si la distribution de ces variables n´est pas normale). Nous avons décrit les variables qualitatives en calculant des fréquences absolues (nombre) et des fréquence relatives (pourcentages). Nous avons utilisé la méthode de standardisation directe (méthode de la population type) pour comparer les taux de mortalité entre les populations des 24 gouvernorats de la Tunisie en tenant compte de la différence de la structure d´âge dans ces populations. La population tunisienne a été utilisée comme population de référence. Pour comparer la mortalité spécifique par COVID-19 entre la Tunisie et les différents pays nous avons utilisé la méthode de standardisation indirecte (méthode de la mortalité type) en prenant la population tunisienne comme population de référence puisque nous ne disposons pas de la répartition des décès COVID-19 par classe d´âge dans les autres pays. Le nombre cumulé observé des décès COVID-19 par pays a été obtenue à partir des données publiées par l´OMS [6].

### Définition des indicateurs de mortalité utilisés

**Taux brut de mortalité par COVID-19 (pour 100 000 habitants) (**TBM**)**: TBM= (Nombre de nouveaux décès COVID-19 durant la période d´étude/la taille de la population au début de l´année 2020) *100 000.

**Taux standardisé de mortalité selon l´âge (**TSM**)** (pour 100 000 habitants) selon la méthode de standardisation directe: TSM=∑_n_i=1 (Ti * proportion de la classe d´âge i dans la population de référence) avec Ti= (nombre de nouveaux décès dans la classe d´âge i/population totale de la classe d´âge i)*100 000.

**Létalité (Case fatality ratio) (%):** (Nombre de décès COVID-19/nombre de cas incidents COVID-19)*100.

**Mortalité proportionnelle à l´épidémie COVID-19 (%):** (Nombre de décès COVID-19/Nombre de décès toutes causes confondues) *100. Coefficient de variation hebdomadaire (CV)(%): c´est le nombre observé S_n_moins le nombre observé en S_n-1_divisé par le nombre de décès en S_n-1_: (S_n_-S_n-1_)/S_n-1_.

**Comparative Morbidity Figure (CMF)**= TSM_gouvernorat A_/TSM_gouvernorat B (le plus faible)_

**Standardized Mortality Ratio(SMR)**: c´est le rapport du nombre observé sur le nombre attendu de décès COVID-19. SMR=M/E avec E =∑_i_i=1PAi*Ti et Ti correspond au taux de mortalité spécifique de la population de référence (population tunisienne).

**Risque Relatif (RR)**: pour mesurer la force d´association entre l´âge et le genre et le risque de décès par COVID-19. RR=RIE+/RIE- avec RIE+: le risque individuel chez les exposés et RIE-: le risque individuel chez les non exposés. L´intervalle de confiance à 95% (IC à 95%) du RR a été calculé. Les seuils utilisés pour l´évaluation du risque hebdomadaire de mortalité en fonction du TBM/100 000 habitants étaient:

**Nous avons aussi calculé les IC à 95% du CMF et du SMR à l´aide de formules suivantes pour tester leurs significativité par rapport à 1** [20]: Log (CMF)_inf_=Log (CMF)-1,96 *√Var (Log (CMF)) Log (CMF)_sup_=Log (CMF)+1,96 *√Var(Log (CMF)) Avec Var (Log (CMF))=(Var(TMS_A_)/TSM_A_2) + (Var(TSM_B_)/TSM_B_2) CMF_inf_= exp (Log (CMF)_inf_); CMF_sup_= exp(Log (CMF)_sup_).


$${\text{SMR}}_{\inf}=\frac{M}{B}*{\left(1-\frac{1}{9+M}-\frac{z\left(1-\frac{\alpha }{2}\right)}{3+\sqrt{M}}\right)}^{3};{\text{ SMR}}_{\sup}=\frac{M+1}{B}*{\left(1-\frac{1}{9+\left(M+1\right)}+\frac{z\left(1-\frac{\alpha }{2}\right)}{3+\sqrt{M}+1}\right)}^{3}$$

**Considérations éthiques**: la confidentialité des données était respectée. Toutes les données ont été analysées de façon anonyme. L´exploration des données personnelles de façon anonyme dans le contexte de l´épidémie COVID-19 était accordée par l´Instance Nationale de Protection de données personnelles.

**Financement de l´étude:** nous n´avons pas reçu de financement pour le déroulement de cette étude.

## Résultats

Durant la période s´étalant entre 02 mars 2020 et 28 février 2021, 8051 décès liés à la COVID-19 ont été déclarés à l´ONMNE soit une mortalité proportionnelle à la COVID-19 de 10,4% (8051/77666).

**Figure 1:**
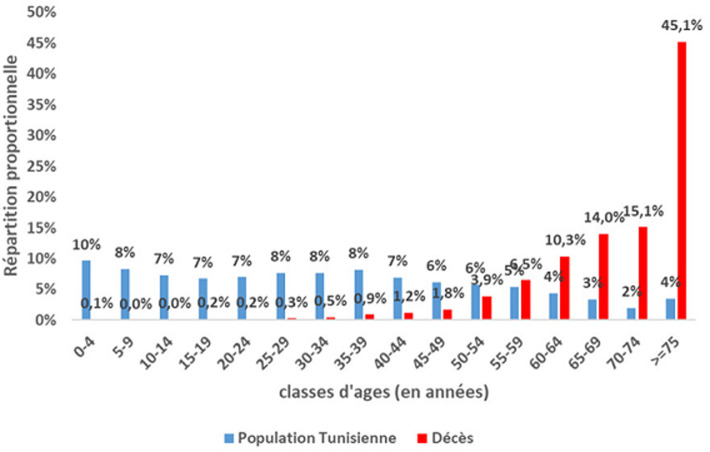
comparaison de la distribution par âge des décès COVID-19 à la population Tunisienne, Tunisie (mars 2020-février 2021)

**Figure 2:**
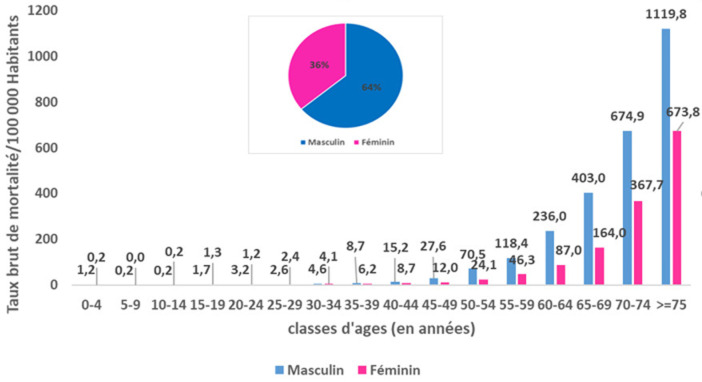
répartition des décès Covid-19 par âge et par sexe, Tunisie (mars 2020-février 2021)

**Caractéristiques sociodémographiques de la population d´étude:** l´âge médian était de 73,0 ans (Intervalle Interquartile [64; 81]): 71,0 ans [63,0-80,0] chez le genre masculin et 75,0 ans [66,0-82,0] chez le genre féminin. Le sex-ratio (M/F) était de 1,8. L´étude de la répartition proportionnelle selon l´âge a montré que 73,7 % des décès COVID-19 sont survenus chez les sujets âgés de 65 ans et plus et 44,8 % chez les sujets âgés de 75 ans et plus (Figure 1). Le RR de décès augmentait progressivement avec l´âge pour atteindre 74,5 (IC à 95 % [60,8-91,4]) chez les sujets âgés de 75 ans et plus par rapport à ceux âgés entre 40 et 45 ans. Les taux bruts de mortalité pour 100 000 habitants étaient de 89,6 et de 48,9 chez les sujets du genre masculin et féminin respectivement soit un RR (M/F) de 1,8; IC à 95% [1,7-1,9]. L´étude du taux de mortalité par âge et par genre a montré que les taux les plus élevés ont été enregistrés chez les sujets de genre masculin âgés de 75 ans et plus (Figure 2). De même, la distribution hebdomadaire de l´âge médian n´a pas montré de différence significative de l´âge de décès en fonction du temps. Concernant la létalité, les proportions les plus élevés étaient enregistrés chez les sujets âgés de 75 ans et plus de genre masculin soit 25,1% versus 16,3% chez les femmes du même âge.

**Caractéristiques cliniques et paracliniques de la population d´étude:** la notion de comorbidité était rapportée dans 25,4% (2049/8051) des cas. Les comorbidités les plus fréquentes étaient: l´hypertension artérielle HTA (57%) et le diabète (53%) (Tableau 1). Quarante-deux pour cent (42%) des décès avaient une seule comorbidité. Hypertension artérielle et diabète était l´association de comorbidité la plus fréquente (59% des cas). Sur l´ensemble des décès déclarés, 6095 (75,7%) ont été confirmés par RT-PCR. Plus de 2 décès sur trois (69 %) ont été diagnostiqués en pré-mortem. Le délai médian entre la date de confirmation et la date de décès était de 6 jours avec un intervalle interquartile de 11 jours.

**Tableau 1:** réparation des décès COVID-19 par comorbidité, Tunisie, mars 2020-février 2021 (n=2036)

Comorbidités	Effectif	Pourcentage (%)
Hypertension artérielle	1161	57,0
Diabète	1074	52,8
Cardiopathie	393	19,5
Insuffisance rénale chronique	200	9,8
Pathologie pulmonaire chronique	151	7,4
Néoplasie	87	4,3
Alzheimer	16	0,8
Autre	452	22,0

**Figure 3:**
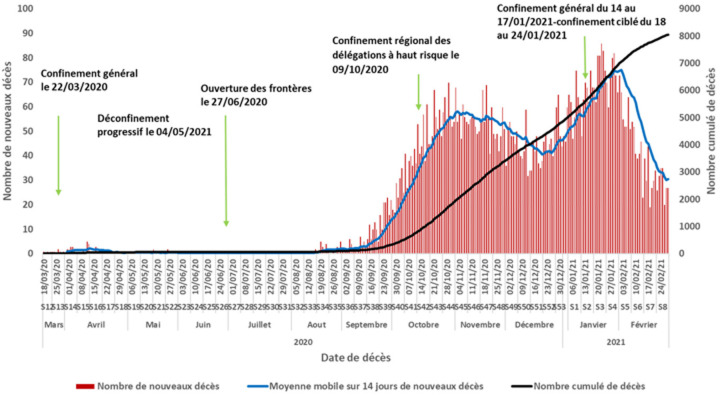
courbe épidémique journalière des décès COVID-19 en Tunisie, mars 2020-février 2021

**Répartition temporelle des décès (courbe épidémique):** l´étude de la distribution journalière du nombre des décès a montré 2 pics en octobre 2020 (S44) et en janvier 2021 (S3) avec respectivement 70 nouveaux décès le 29 octobre 2020 et 86 nouveaux décès le 22 janvier 2021 (Figure 3). De même, la moyenne journalière de décès était de 58 nouveaux décès par jour en S44/2020 (du 26 octobre 2020 au 1^er^ novembre 2020) et de 77 nouveaux décès par jour en S3/2021 du (18 au 24 janvier 2021). Le nombre des décès est passé de 294 en septembre 2020 à 1444 en octobre 2020 pour le 1^er^ pic (CV de 391% en octobre 2020) et de 1402 en décembre 2020 à 2077 en janvier 2021 pour le 2^e^ pic (CV de 48% en janvier 2021). L´évolution hebdomadaire du TBM/100 000 habitants par tranche d´âge a montré que le risque de décès était très faible pour toutes les tranches d´âges jusqu´au début Septembre 2020 (S36). Ce risque a augmenté à partir de cette date et qu´il était le plus important chez les sujets âgés (Annex 1).

**Figure 4:**
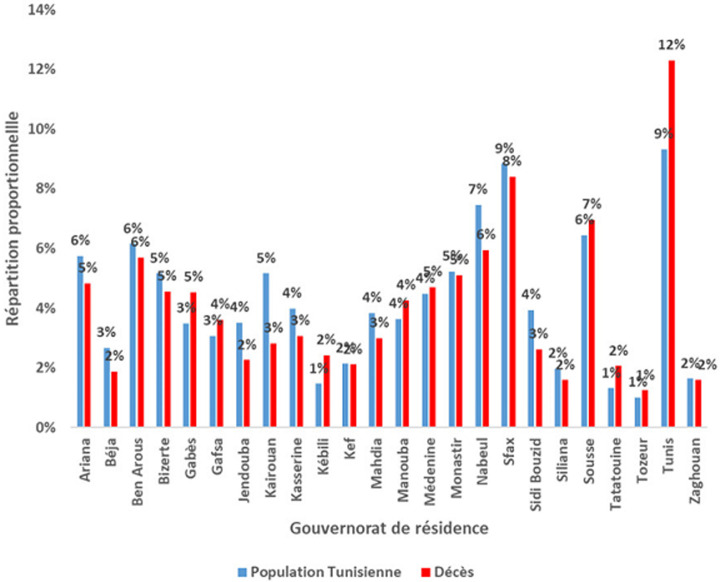
comparaison de la distribution par gouvernorat des décès COVID-19 à la population tunisienne, Tunisie (mars 2020 - février 2021)

**Figure 5:**
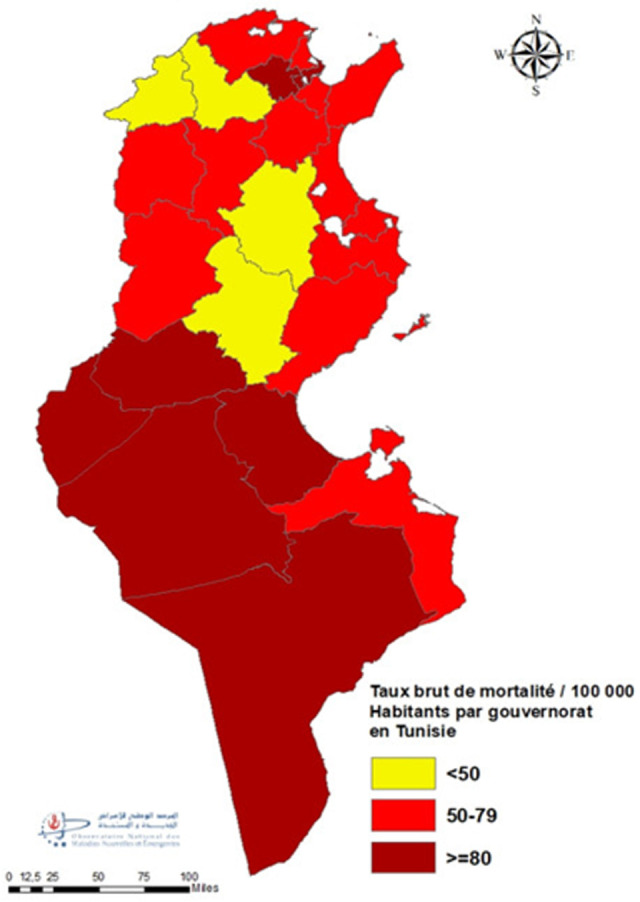
répartition du taux de mortalité brut COVID-19 par gouvernorat de résidence, mars 2020-février 2021

**Figure 6:**
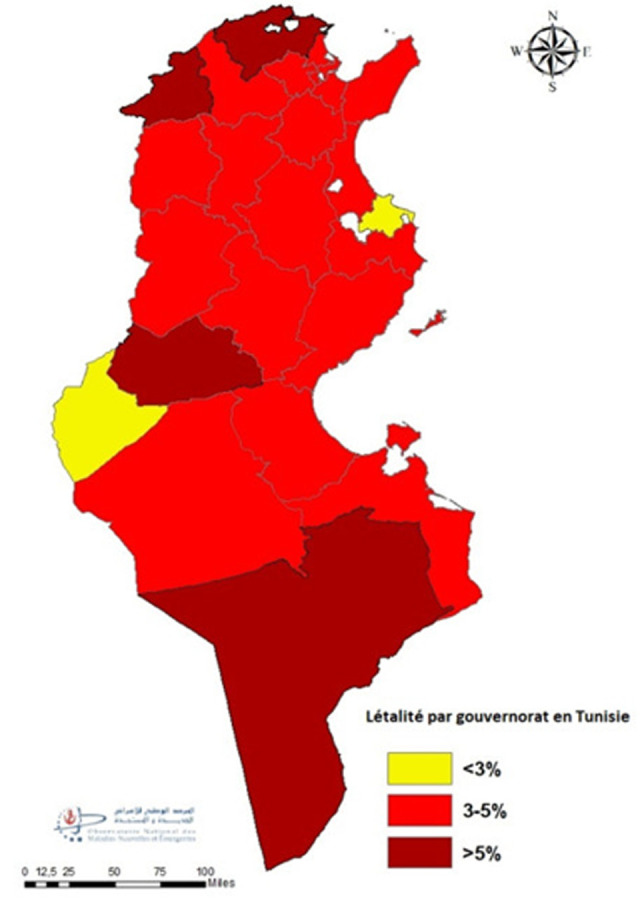
répartition de la létalité COVID-19 par gouvernorat de résidence, mars 2020-février 2021

**Répartition spatiale des décès:** l´étude de la répartition proportionnelle des décès par gouvernorat de résidence a montré que Tunis (12%), Sfax (8%) puis Sousse (7%) étaient les gouvernorats les plus touchés. Les pourcentages les plus faibles étaient enregistrés au niveau de la région Nord-Ouest (Kef (2%), Jendouba (2%) et Béja (2%)) (Figure 4). L´analyse du TBM pour 100000 H par gouvernorat de résidence a montré que 38% (9/24) des gouvernorats ont enregistrés des taux supérieurs au niveau national. Les TBM pour 100 000 habitants les plus élevés étaient observés à Kébili (115,4) suivi par Tataouine (109,8) et Tozeur (87,0). Les gouvernorats de Jendouba et Kairouan ont enregistrés les taux les plus faibles avec respectivement 45,0 et 38,2 (Figure 5). L´évolution hebdomadaire du TBM par gouvernorat a mis en évidence que tous les gouvernorats ont été touchés avec un risque élevé à très élevé entre S44 et S48/2020 avec atteinte marquée pour les gouvernorats du Sud (Kebili, Tataouine et Gafsa) (Annex 2). Par ailleurs, les létalités les plus élevées étaient observées aux gouvernorats de Jendouba (6,8%) et Tataouine (6,5%) et les valeurs les plus faibles étaient observés aux gouvernorats de Monastir (2,9%) et Tozeur (2,0%) (Figure 6).

**Répartion des décès COVID-19 par lieu de décès:** la majorité des décès sont survenus dans des structures hospitalières (88,3%) dont 86% au niveau du secteur public. L´établissement public de santé (EPS) était la structure hospitalière public dans laquelle est survenu plus de la moitié des décès (58%).

**Standardisation directe:** les CMF ont été calculés par rapport au TSM de Jendouba qui est considéré le plus faible (34,9/100 000 habitants). Les TSM pour 100 000 habitants les plus élevés ont été enregistrés dans la région du Sud tunisien: les gouvernorats de Kébili (114,9), Tataouine (109,9) et Tozeur (87,4) avec respectivement des CMF par rapport au TSM de Jendouba de 3,8 (IC à 95% [2,7-4,0]), 2,8 (IC à 95% [2,3-3,5]) et 2,8 (IC à 95% [2,3-3,5]). Les TSM pour 100 000 habitants les plus faibles ont été observés dans la région du Nord-Ouest: Siliana (44,5), Béja (39,0) et Kairouan (38,5) avec respectivement des CMF de 1,3 (IC à 95% [1,0-1,6]), 1,1 (IC à 95% [0,9-1,4]) et 1,1 (IC à 95% [0,9-1,3]) (Tableau 2).

**Tableau 2:** répartition du taux standardisé de mortalité par COVID-19 par gouvernorat de résidence en Tunisie, mars 2020-février 2021

Gouvernorat de résidence	Taux de mortalité*			
	Brut	Standardisé	CMF**	Intervalle de confiance à 95%
Kébili	116,0	114,9	**3,3**	[2,7-4,0]
Tataouine	109,9	98,0	**2,8**	[2,3-3,5]
Tozeur	87,4	96,4	**2,8**	[2,2-3,5]
Sousse	76,0	89,7	**2,6**	[2,2-3,0]
Manouba	82,3	87,9	**2,5**	[2,1-3,0]
Gafsa	82,5	86,9	**2,5**	[2,1-3,0]
Gabès	91,0	86,9	**2,5**	[2,1-3,0]
Tunis	92,3	86,1	**2,5**	[2,1-2,9]
Monastir	68,8	83,1	**2,4**	[2,0-2,8]
Ben Arous	65,1	76,0	**2,2**	[1,8-2,6]
Ariana	59,3	73,7	**2,1**	[1,8-2,5]
Zaghouan	68,1	70,3	**2,0**	[1,6-2,5]
Médenine	73,9	68,9	**2,0**	[1,7-2,4]
Sfax	66,7	65,7	**1,9**	[1,6-2,2]
Kasserine	53,7	59,7	**1,7**	[1,4-2,1]
Nabeul	56,0	58,6	**1,7**	[1,4-2,0]
Bizerte	61,9	57,9	**1,7**	[1,4-2,0]
Mahdia	54,6	54,8	**1,6**	[1,3-1,9]
Kef	68,9	51,3	**1,5**	[1,2-1,8]
Sidi Bouzid	46,4	47,3	**1,4**	[1,1-1,7]
Siliana	56,2	44,5	**1,3**	[1,0-1,6]
Béjà	49,1	39,0	**1,1**	[0,9-1,4]
Kairouan	38,3	38,5	**1,1**	[0,9-1,3]
Jendouba	45,0	34,9	**REF**	
Tunisie	69,1	62,9****		

* TM/100 000 Habitants ** Standardisation directe en prenant la population tunisienne (INS 2019) comme population de référence ***CMF (Comparative Morbidity Figure) =TSM gouvernorat /TSM le plus faible (Jendouba) **** Standardisation sur la population mondiale OMS (2002-2025)

**Standardisation indirecte:** l´étude du SMR a montré un risque de décès significativement plus élevé avec une surmortalité par rapport à la Tunisie pour les pays suivants: le Mexique (SMR=2,506; IC à 95% [2,495-2,518]), l´Italie (SMR=2,36; IC à 95% [2,34-2,37]) et l´Afrique du Sud (SMR=1,84; IC à 95% [1,83-1,86]). D´autre part, le SMR était significativement plus faible par rapport à la population tunisienne pour les pays de l´Asie: une sous mortalité a été observé pour la Chine (SMR=0,00042; IC à 95% [0,00041-0,00043]), le Vietnam (SMR=0,0006; IC à 95% [0,0004-0,0008]), le Singapour (SMR=0,006; IC à 95% [0,004-0,008]) et l´Inde (SMR=0,0220; IC à 95% [0,0219-0,0221]) ainsi que pour les pays de l´Océanie: l´Australie (SMR=0,033; IC à 95% [0,031-0,035]) et la Nouvelle Zélande (SMR=0,005; IC à 95% [0,003-0,007]).

## Discussion

La présente étude a mis en évidence la sévérité de l´épidémie dans notre pays pendant la période d´étude s´étalant de S9-2020 à S8-2021 avec un taux national de mortalité de 68,8/100 000 habitants, une létalité de 3,5% et des disparités régionales affectant le plus la région du Sud Tunisien (Kébili, Tataouine et Tozeur). Au terme de cette étude descriptive, les groupes à risque de décès COVID-19 étaient les sujets âgés du genre masculin, ayant des comorbidités (HTA et diabète en particulier), résidaient dans la région du Sud Tunisien Les comparaisons internationales par standardisation indirecte par rapport à la population tunisienne de référence ont montré une sous mortalité marquée pour les pays de l´Asie (la Chine, le Vietnam, le Singapour et l´Inde) et une surmortalité principalement au niveau du Brésil, l´Afrique du Sud, le Mexique et l´Italie.

**Points forts de l´étude:** notre étude est la première à l´échelle nationale qui s´est intéressée à l´étude de la mortalité par COVID-19 depuis le début de l´épidémie en Tunisie sur une période d´une année. La mortalité est un indicateur objectif permettant d´évaluer l´état de santé de population, la qualité de prise en charge des cas COIVD-19 et les performances du système de santé orientant ainsi les décisions en santé publique en fonction des priorités [21-23]. Les principaux indicateurs de mortalité ont été étudiés à travers ce travail. Le caractère prospectif de l´étude a permis le suivi des cas COVID-19 aussi bien probables que confirmés dont l´évolution était marquée par le décès. Ceci permet la mesure directe de la mortalité et la suspicion des facteurs prédictifs de mortalité grâce à la temporalité de l´association entre les expositions et le décès.

Le système de surveillance de mortalité, à la fois actif et passif, a permis de croiser plusieurs sources de déclaration afin de s´assurer de la validité et de la complétude des données individuelles collectées de façon continu. Cette méthode a contribué à l´augmentation de la sensibilité (moins de sous déclarations), à la représentativité et à l´exhaustivité du système de surveillance [16]. De même, la définition du décès COVID-19 ainsi que la déclaration de l´information étaient simples et standardisée réduisant ainsi le biais de mesure et favorisant la réactivité du système. Ce système a permis aussi de disposer d´indicateurs épidémiologiques de description et de suivi de la pandémie en Tunisie en terme du temps (courbe épidémique), d’espace (cartographie) et de personnes (caractéristiques socio-démographiques et cliniques). Globalement, le système de surveillance mis en place remplis les critères de qualité requises (sensibilité, flexibilité, réactivité, représentativité, simplicité et acceptabilité). Ceci permet ainsi la formulation de conclusions et de recommandations et la retro information aux intervenants d´action en santé publique. De ce fait, c´est un système qui a permis de répondre aux 3 objectifs principales d´un système de surveillance épidémiologique: décrire, alerter et évaluer.

La méthode de standardisation consiste à prendre en compte, lors des comparaisons entre les populations, les différences de distribution de facteurs connus ou facteurs de confusion (essentiellement l'âge dans cette étude) pouvant influencer l'événement mesuré (la mortalité). C'est-à-dire que la mortalité standardisée permet de corriger les éventuelles variations dues au changement de la pyramide des âges entre les gouvernorats d´une part et entre les pays d´autre part. L´effet de l´âge est ainsi neutralisé en utilisant la population tunisienne comme population de référence ce qui limite le biais de confusion [23,24].

**Limites de l´études:** malgré les points forts, certaines limites comme tout travail sont à citer: l´absence d´un identifiant unique pour chaque patient, les déclarations tardives des décès ainsi que l´absence d´un système d´information de collecte de données auprès des différentes sources d´information au niveau central. Ces limites ont engendré un retard de notification et d´investigation des décès, un délai important pour la consolidation des données et la vérification des doublons. De même, la mortalité COVID-19 pourrait être sous-estimée devant la non inclusion des sujets ayant présenté des formes asymptomatiques ou légèrement symptomatique et qui n´ont pas étaient testés entrainant un biais de mesure. Ce biais peut être limité par la confirmation en post mortem des décès cliniquement suspects. Le caractère émergent du virus SARS-Cov-2 et le non recul suffisant de la littérature par rapport aux complications possibles de la maladie (Syndrome post-COVID) pourraient être une source potentielle de biais de mesure par la sous-estimation de la mortalité proportionnelle à la COVID-19 [25,26]. Le dynamisme de la définition de décès COVID-19 qui est actualisée et adaptée aux nouvelles connaissances scientifiques internationales a permis de réduire ce biais. L´étude de mortalité est ainsi influencée par la qualité des certificats médicaux de décès qui malgré l´amélioration comparativement aux années précédentes, reste insuffisante et ne satisfait pas le modèle requis avec la mention de 3 causes de décès: immédiate, initiale et associée. L´exhaustivité de la notification des décès est aussi un élément important à considérer [27]. La mortalité proportionnelle à la COVID-19 pourrait par ailleurs être sous-estimée en présence de comorbidités rendant l´attribution directe du décès à la COVID-19 difficile [18]. Le système de surveillance rapide de la mortalité dans le contexte de l´épidémie permet de pallier en partie à ces insuffisances. Cependant, il est important de souligner que la mortalité ne mesure que partiellement l´impact sanitaire de cette épidémie. D´autres dimensions ainsi utiles non pas été étudiées tels que le nombre de malades atteint de formes sévères (décès en fonction de la présentation clinique de la maladie), le taux d´hospitalisations, le séjour en soins intensifs reflétant ainsi la charge lourde sur nos institutions sanitaires avec des capacités hospitalières limitées [28].

**Caractéristiques épidémio-cliniques des décès COVID-19:** l´analyse des données par âge et par genre a permis de conclure à une prédominance masculine; Prés de 2 décès du genre masculin pour un décès du genre féminin. De même, toutes les classes d´âges ont été touchées et particulièrement les sujets âgés de 75 ans et plus. L´atteinte fréquente des sujets âgés du genre masculin a été également rapportée par d´autres études [29-31]. A l´échelle régionale, des disparités au niveau du taux de mortalité ont été constatées malgré une diffusion géographique large de l´épidémie sur le territoire national avec une transmission communautaire soutenue stade 4 [32]. Les gouvernorats de Kébili, Tataouine et Tozeur ont été les plus impactés par l´épidémie COVID-19 avec des taux bruts de mortalité près du double de la moyenne nationale. Cette disparité régionale a été également observée au niveau de la létalité: les gouvernorats de Jendouba et Tataouine ont enregistré les proportions les plus élevées soit le double de la létalité nationale. Par contre les pourcentages les plus faibles ont été constatés à Monastir et Tozeur. Cela pourrait s´expliquer par la variation de l´activité du dépistage d´une part et à la rapidité et la qualité de prise en charge des cas COVID-19 d´autres part en rapport avec la proximité à des services de prise en charge adéquats. En comparaison à la distribution proportionnelle de la population tunisienne par âge, la distribution des décès COVID-19 était différente avec des valeurs plus faibles pour les âges jeunes et qui commençait à s´inverser à partir de l´âge de 60 ans pour devenir manifeste chez les sujets âgés de 75 ans (45% pour la population d´étude versus 4% pour la population tunisienne) (Figure 1). La distribution proportionnelle par genre a montré que la prédominance masculine des décès COVID-19 n´est pas reflétée au niveau de la population générale: 64,0% versus 49,6% et 36,0% versus 50,4% respectivement pour le genre masculin et féminin (Figure 3) [17].

**Figure 7:**
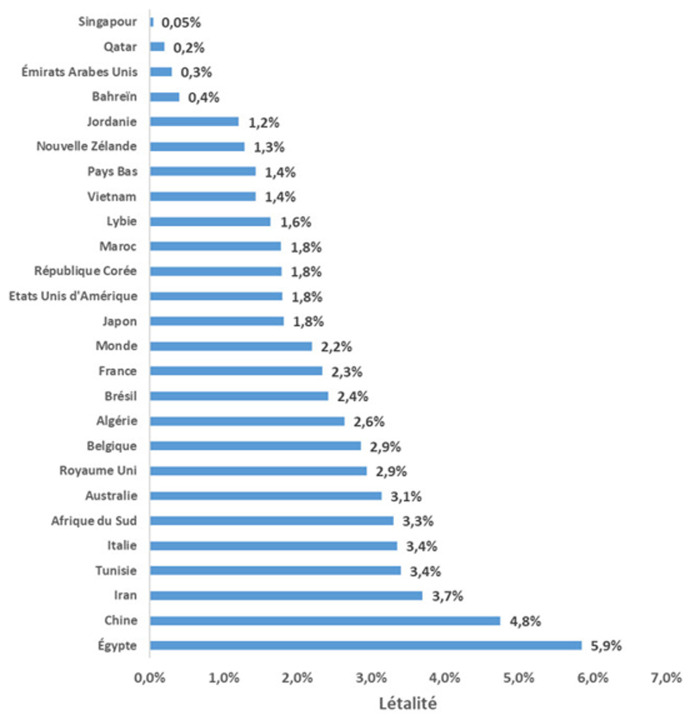
répartition du taux de la létalité COVID-19 dans le monde dépuis le début de la pandémie à la date du 28 février 2021 [7]

Quant à la distribution proportionnelle par gouvernorat de résidence, la distribution des décès COVID-19 était similaire à l´exception du gouvernorat de Tunis (12% pour la population d´étude versus 9% pour la population tunisienne). Ceci pourrait être due à la concentration des structures sanitaires publiques et privés au niveau de la capitale Tunis [33]. La létalité nationale était plus élevée que la létalité mondiale. En comparaison avec d´autres pays, la Tunisie se situe en position intermédiaire entre les pays à faible létalité (Singapour, Qatar, Émirats Arabes Unis et Bahreïn) et les pays à forte létalité (Iran, Chine et Égypte) tout en étant plus proche des pays à forte létalité. Le niveau de létalité était par ailleurs proche de celui l´Italie, l´Australie et l´Afrique du Sud (Figure 7). En effet, la létalité est un indicateur reflétant la sévérité de l´épidémie, est très influencée par le dépistage qui reste très faible en Tunisie comparativement à d´autres pays [34]. Lors de la première phase de la pandémie en Tunisie, nous n´avons pas enregistré un nombre important de décès avec un impact faible sur les capacités du système de santé. Ceci grâce aux mesures préventives qui ont été prises très précocement dès les premiers cas enregistrés tels que la fermeture des écoles, le confinement général et la fermeture des frontières.

Les deux pics de décès observés au cours de la 2^e^ phase de la première seraient essentiellement liés au relâchement des mesures barrières, au défaut d´application des protocoles sanitaires dans les différents secteurs ainsi qu´à l´absence de contrôle des mesures qui ont été prises (déplacement entre les gouvernorats, maintien des manifestations avec rassemblements). Ainsi le changement climatique pourrait expliquer en partie cette recrudescence: une relation non linéaire a été démontrée entre les décès COVID-19 et la température à Wuhan, d´autre part le risque quotidien de décès COVID-19 diminue de 12,3% (IC à 95% [3,4-20,4%]) pour chaque augmentation de 1,0^°^C de la température. De même, une baisse significative du nombre quotidien de cas et de décès COVID-19 dans les pays les plus chauds et les moins humides du monde (Ghana, Oman et Inde) a été mise en évidence: le nombre de décès COVID-19 est réduit de ß= -0,187 (p=0,004) pour une augmentation de 1% de l´humidité et ß= -1,35 (p<0,001) pour une augmentation de 1% de température [35-37]. Par ailleurs, les comorbidités les plus fréquentes dans notre population d´étude étaient l´hypertension artérielle, le diabète et les cardiopathies ce qui est fréquemment rapporté dans la littérature [29,30]. D´autres facteurs comme l´obésité et le tabac ont été rapportés par littérature comme facteurs de risque mais non étudiés à travers ce travail pour éviter la complexité de l´outil de collecte de données [22,38]. Ces comorbidités ont été également admises dans la littérature comme principaux facteurs de risque de mortalité en cas d´infection humaine par les autres coronavirus (SARS-Cov et MERS-Cov) [31,39,40]. Ceci pourrait être expliqué par l´effet de l´âge avancé et les pathologies qui lui sont alors associés.

**Mortalité toutes causes confondues:** les sujets âgés du genre masculin particulièrement touchés par la mortalité COVID-19 ont été aussi touchés avec prédilection par la mortalité toutes causes confondues en Tunisie durant les dernières décennies [18]. La surmortalité constitue un bon indicateur de l'impact global du Covid-19. C´est un indicateur plus fiable que le décompte du nombre de décès attribuables à la COVID-19 [28]. Au niveau national, le nombre de décès toutes causes confondues durant l´année 2020 était de 75365 décès, soit un accroissement de 5,3% ou 3764 décès de plus qu´en 2019 selon une étude publiée par l´INS le 10 mars 2021 [18]. L´augmentation de décès sur l´année s´étalant entre mars 2020 à février 2021 est estimé à près de 4850 morts et ne peut être considérée comme significative ni associée aux explications conventionnelles liées à la saisonnalité de la mortalité. En effet, les mesures prises de restriction et d´hygiène respiratoire pourraient réduire les décès habituels par accident de la voie publique et par infections respiratoires virales ou bactériennes [18]. En comparaison avec d´autres pays, un excès de mortalité par rapport à l´attendu lié à la pandémie COVID-19 a été observé dans les États-Unis d´Amérique et dans certains pays de l´Europe [28,41,42]. En Chine, l´excès de mortalité a été uniquement marqué à Wuhan (région centrale épicentre de l´épidémie) pendant la période du 1^er^ janvier à 31 mars 2020 avec un surcroît de 42% [43]. Ces résultats sont à interpréter avec précautions vu la différence des méthodologies utilisées pour l´étude de la surmortalité.

### Standardisation

**Directe:** à l´échelle nationale, les gouvernorats qui ont enregistrés les taux standardisés les plus élevés (Kébili, Tataouine et Tozeur) avec des CMF respectifs de 3,8, 2,8 et 2,8 par rapport au gouvernorat de Jendouba qui a enregistré le taux standardisé le plus faible. L´enregistrement des TSM plus élevés dans certains gouvernorats par rapport aux autres pourraient être expliqué par la précarité de l´infrastructure sanitaire ne permettant pas une prise en charge adéquate et précoce des cas ainsi que par le déficit en ressources humaines en termes de médecins réanimateurs.

**Indirecte:** à l´échelle internationale, la Tunisie a présenté globalement un risque de mortalité supérieur à celui des pays de l´Europe, de l´Asie (Chine, Inde, Vietnam, Japon, Singapour et pays du Golf) et de l´Océanie (Australie et Nouvelle Zélande) (Tableau 2). Nous avons marqué un risque de mortalité inférieur par rapport à la Jordanie, l´Iran, pays de l´Amérique (Brésil, Mexique et États-Unis). Cette sous mortalité par rapport à la population tunisienne pourrait être expliquée en partie par la robustesse de notre système de surveillance épidémiologique qui est très sensible et par le relâchement de l´application des mesures sociales de santé publique et le défaut de mise en œuvre des protocoles sanitaires en particulier à partir de la 2^e^ phase.

## Conclusion

Tous les indicateurs étudiés ont mis en évidence la sévérité de l´épidémie dans notre pays pendant la première année de la pandémie (mars 2020-février 2021) avec des disparités régionales affectant le plus la région du Sud. Les conséquences de cette pandémie auraient été encore plus dramatiques en l´absence des mesures adéquates qui ont été prises précocement (confinement général, fermeture des frontières, couvre-feu, protocoles sanitaires dans les différents secteurs, confinement des délégations à haut risque) et dynamiques selon l´évolution de la situation épidémiologique. Le respect des mesures barrières (port de masques, hygiène des mains et la distanciation physique), l´application effective des protocoles sanitaires mis en place au niveau des différents secteurs ainsi que la précocité de prise en charge adéquate des cas demeurent les piliers de la lutte contre cette épidémie en association avec la vaccination de la population au cours des prochains jours. Le renforcement de la surveillance et de la vigilance sanitaire en particulier face à l´émergence de nouvelles variantes dans certains pays (Royaume Uni-Brésil et Afrique du Sud) serait indispensable. A la lumière des résultats de cette étude et dans la perspective de nouvelles poussées épidémiques possibles de la pandémie surtout avec l´émergence de variants préoccupants (VOC) en début de l´année 2021, des études qualitatives de type CAP sur le déroulement de la pandémie ainsi que les mesures mises en place (y compris la vaccination anti-COVID-19) pour freiner son extension sont recommandées.

### Etat des connaissances sur le sujet

- *La COVID-19 est une pandémie, tous les pays du monde ont été affectés;*
- *Les sujets à risque de décès sont les sujets âgés de sexe masculin avec comorbidités*.

### Contribution de notre étude à la connaissance

- *L´intérêt d´application précoce des mesures non pharmaceutiques de santé publique pour réduire l´impact de la pandémie COVID-19;*
- *L´intérêt de la surveillance épidémiologique pour suivre le dynamisme de l´épidémie et aider à la prise de décision en santé publique;*
- *L´intérêt de croisement de données de plusieurs sources d´information pour améliorer la notification des décès*.

## Remerciements

Nous tenons à remercier toute l´équipe de terrain, les équipes de réponse rapide et les équipes de directions régionales de santé ainsi que toutes les structures sanitaires publiques et privées pour les efforts fournis au cours de cette épidémie.

## Annexes

**Annex 1**: répartition hebdomadaire du taux brut de mortalité par groupe d´âge, Tunisie, mars 2020-février 2021

**Annex 2**: répartition hebdomadaire du taux brut de mortalité par gouvernorat, Tunisie, mars 2020-février 2021
